# Extract of *Cimicifuga racemosa (L.) Nutt* protects ovarian follicle reserve of mice against *in vitro* deleterious effects of dexamethasone

**DOI:** 10.1590/1414-431X2023e12811

**Published:** 2023-09-22

**Authors:** E.I.T. de Assis, V.A.N. Azevedo, M.F. de Lima, F.C. Costa, L.R.F.M. Paulino, P.A.A. Barroso, M.H.T. Matos, A.P.O. do Monte, M.A.M. Donato, C.A. Peixoto, A.N. Godinho, J.M.O. Freire, A.L.P. Souza, J.R.V. Silva, A.W.B. Silva

**Affiliations:** 1 Laboratório de Biotecnologia e Fisiologia da Reprodução, Universidade Federal do Ceará, Sobral, CE, Brasil; 2 Núcleo de Pesquisa em Experimentação Animal, Universidade Federal do Ceará, Sobral, CE, Brasil; 3 Núcleo de Biotecnologia Aplicada ao Desenvolvimento do Folículo Ovariano, Universidade Federal do Vale do São Francisco, Petrolina, PE, Brasil; 4 Laboratório de Ultraestrutura, Centro de Pesquisas Aggeu Magalhães (CPqAM)/FIOCRUZ, Universidade Federal de Pernambuco, Recife, PE, Brasil

**Keywords:** Folliculogenesis, Toxicity, Mice, Glucocorticoid, *In vitro* culture

## Abstract

The present study aims to investigate if *Cimicifuga racemosa (L.) Nutt* extract (CIMI) reduces deleterious effects of dexamethasone (DEXA) in ovaries cultured *in vitro*. Mouse ovaries were collected and cultured in DMEM^+^ only or supplemented with 5 ng/mL of CIMI, or 4 ng/mL DEXA, or both CIMI and DEXA. The ovaries were cultured at 37.5°C in 5% CO_2_ for 6 days. Ovarian morphology, follicular ultrastructure, and the levels of mRNA for *Bax, Bcl-2,* and *Caspase-3* were evaluated. The results showed that DEXA reduced the percentage of morphologically normal follicles, while CIMI prevented the deleterious effects caused by DEXA. In addition, DEXA negatively affected the stromal cellular density, while CIMI prevented these adverse effects. Ovaries cultured with DEXA and CIMI showed similar levels of mRNA for *Bax, Bcl-2*, and *Caspase-3* compared to those cultured in control medium, while ovaries cultured with DEXA had increased expression of the above genes. Additionally, the ultrastructure of the ovaries cultured with CIMI was well preserved. Thus, the extract of CIMI was able to prevent the deleterious effects caused by DEXA on cultured mouse ovaries.

## Introduction

Glucocorticoids (GCs) are steroid hormones widely used in intensive care due to their anti-inflammatory and immunosuppressive effects. Among exogenous GCs, hydrocortisone is identical to endogenous cortisol, while prednisone, prednisolone, dexamethasone (DEXA), and others are synthetic and vary in potency and affinity for their receptor. DEXA is a potent drug that has been widely used by the general population, including women in reproductive age. Recent studies have shown satisfactory DEXA results in the treatment of acute respiratory distress syndrome caused by SARS-CoV-2 (COVID-19) infection (1-3). *In vitro*, DEXA (4 ng/mL) suppressed IL-6 production in normal human bronchial epithelial (NHBE) cells stimulated by TNF-α (4).

Although effective, long-term exposure to GCs leads to numerous changes in several biological functions, including the reproductive system (5). Glucocorticoid receptors (GR) are expressed in many tissues (6), including the ovaries (7). Dare et al. (8) found that high doses of DEXA (10 and 12 mg/kg) cause multiples changes in the histological characteristics of the ovary and uterus. In follicles at the earliest stages of development, DEXA increased the rate of degeneration in fragments of ovarian tissues cultured *in vitro* for six days (9). Previously, Van Merris et al. (10) showed that DEXA impairs follicle and oocyte maturation by activating steroidogenesis pathways and plays a negative role in mouse ovarian function and in the development and pre-implanted embryos. DEXA acts by suppressing the expression of StAR protein induced by luteinizing hormone (LH) and the production of progesterone by a mechanism mediated by GR (11). In another study in mice, maternal exposure to DEXA led to a significant reduction in the pool of healthy primordial follicles and increased follicular atresia in the offspring (5). Studies to investigate the potential of natural substances capable of attenuating the deleterious effects of GCs on ovarian function in order to preserve fertility are extremely necessary. Our recent results showed that *Cimicifuga racemosa* (L.) Nutt (CIMI) extract protects ovarian follicles from atresia induced by doxorubicin in cultured ovaries (12).

CIMI is a widely used plant that is effective in relieving the symptoms of menopause. It is a member of the *Ranunculaceae* family, native to eastern North America (13). Phytochemical studies have shown that constituents of CIMI include triterpene glycosides, phenolic constituents, and formonectin (an isoflavone). Among the biological activities associated with CIMI, antioxidant, anti-inflammatory, antidiabetic, antiviral, antiangiogenic, vasodilatory, and immunosuppressive effects are highlighted (14). The ethanolic and isopropanolic extracts were equipotent to the action of estrogenic and dopaminergic drugs (15). Azouz et al. (16) reported that CIMI ethanolic extract inhibited androgen aromatization in rats with letrozole-induced polycystic ovary syndrome (PCOS), compensating the oxidative ovarian stress, which may be involved in the pathogenesis of PCOS. However, it is still unknown whether CIMI extract has a protective effect against DEXA-induced damage in the mouse ovary.

Our study aimed to investigate the CIMI extract action against the deleterious effects of DEXA on morphology, activation, growth, organization of extracellular matrix (ECM), density of cells in ovarian stromal tissue, expression of mRNA for C*aspase-3, Bax,* and *Bcl-2*, and the ultrastructure in mouse ovaries cultured *in vitro.*

## Material and Methods

### Chemicals

Dexamethasone (CAS number: 50-02-2), *Cimicifuga racemosa* extract (CAS number: 8477626-1, Sigma), the culture medium and other chemicals used in this study were purchased from Sigma Chemical Co. (USA), unless otherwise indicated. *Cimicifuga racemosa* powdered extract was dissolved in 60% (v/v) ethanol for all experiments (17).

### Animals and evaluation of estrous cycle

For this study, Swiss mice (*Mus musculus*) were kept in polyethylene boxes lined with wood shavings (6 animals/box), with free access to filtered water and feed. The animals were kept at an average temperature of 22±2°C, following 12 h light/dark cycles. The animals were used according to the guidelines and normative resolutions of the National Council for Control in Animal Experimentation (CONCEA, Brazil). This study was approved by the institution's Ethics Committee on the Use of Animals (CEUA), approved under protocol No. 05/18.

All female mice of 18 g and/or 2 months of age had their estrous cycle evaluated once a day for 15 days between 9:00 and 10:00 a.m. by a single evaluator, as established by Marcondes et al. (18). Cycle stage, i.e., proestrus, estrus, metestrus, or diestrus was determined according to the observed cells. Only females with a regular cycle lasting 4 to 5 days were used to carry out the experiment. Animals with irregular estrous cycle were excluded from the experiment.

### Experimental design

Ovaries (n=64) were collected from euthanized mice and cultured individually in 24-well plates containing 1 mL DMEM/HAMS F12 supplemented with ascorbic acid (10 μg/mL), penicillin G (75 µg/mL), ITS (10 μg/mL insulin, 5.5 μg/mL transferrin, and 5 ng/mL selenium), and bovine serum albumin (BSA, 10 μg/mL), according to the protocol described by O'Brien et al. (19). This base medium was called DMEM^+^. Then, the ovaries were cultured in DMEM^+^ alone or supplemented with 5 ng/mL of CIMI, 4 ng/mL of DEXA, or both 5 ng/mL CIMI and 4 ng/mL DEXA. The culture was performed at 37.5°C in 5% CO_2_ for 6 days (12). Approximately half of the culture medium was replaced every 2 days. The concentration of CIMI used in this study was chosen based on previous experiments of our group (12), while the concentration of DEXA (4 ng/mL) was defined based on previous *in vitro* studies (4,20). At the end of the culture period, for each treatment, eight ovaries were fixed for histological analysis, four ovaries were used for transmission electron microscopy analysis, and four ovaries were stored at -80°C for RT-qPCR.

### Morphological assessment of ovarian follicles and evaluation of cellular density in ovarian stroma

Cultured ovaries were fixed in paraformaldehyde (4% in phosphate buffered saline - PBS, pH 7.4) for 24 h and processed for classical histology, according to methodology described by Illera et al. (7). After fixation, the ovaries were dehydrated in gradual series of ethanol, clarified with xylene, and embedded in paraffin wax. For each ovary, 7-µm sections were mounted on slides and stained using the hematoxylin-eosin (HE) method. Quantitative analysis of slides was carried out by an experienced researcher who was unaware of the treatment of each group under analysis. Follicle population was counted in every third section of the ovaries and the follicles were classified as primordial, primary, and secondary follicles based on their morphological appearance, according to Lins et al. (21). These follicles were individually classified as morphologically normal when an intact oocyte was surrounded by granulosa cells well organized in one or more layers, and which had no pyknotic nucleus. Degenerated follicles were defined as those with a retracted oocyte, pyknotic nucleus, and/or surrounded by disorganized granulosa cells, detached from the basement membrane (22).

The evaluation of stromal cell density in ovarian tissues was performed after 6 days of culture in the different treatments. For each treatment, twenty random fields from different sections were evaluated using a camera attached to a microscope (Nikon, Eclipse, TS 100, Japan), and the images were analyzed by ImageJ Software (version 1.51p, 2017, NIH, USA). The number of stromal cells was manually counted in an area of 100 μm^2^ as described previously (23). All evaluations and measurements were performed by a single operator.

### Analysis of the extracellular matrix

To assess the collagen fibers of the ECM in ovarian cortex, staining with picrosirius red (Abcam Kit, UK) was performed following the methodology described by Rittié (24) with modifications. Ovarian sections of 7 µm were dewaxed in xylene and incubated in Sirius red solution (0.1%) for 1 h at room temperature. Then, the excess dye was removed with acetic acid solution (0.5%) and the sections were dehydrated and subjected to slide assembly with subsequent observation under an optical microscope (Nikon, Eclipse, TS 100). For each treatment, the percentage of the area occupied by collagen fibers in ten different fields was measured with the aid of a DS Cooled DS DS-Ri1 camera attached to a microscope (Nikon, Eclipse, TS 100), and the images were analyzed by ImageJ Software (version 1.51p, 2017) with 400× magnification. Only collagen fibers were marked in red with the picrosirius color, while the follicles remained colorless. The software automatically excludes the circumference of unstained follicles from the total area marked in red.

### RNA isolation and real time quantitative PCR (qPCR)

After 6 days of culture, the ovaries of animals from each treatment were collected and stored at −80°C until total RNA extraction for further analysis of mRNA levels for *Bax, Bcl-2*, and *Caspase-3*. Total RNA extraction was performed using a TRIzol¯ purification kit (Invitrogen, Brazil) according to the manufacturer's instructions. The mRNA quantification was performed using SYBR Green. PCR reactions were composed of 1 μL of cDNA as a template in 7.5 μL of SYBR Green Master Mix (PE Applied Biosystems, USA), 5.5 μL of ultra-pure water, and 0.5 μM of each primer. The primers are designed to amplify *Bax*, *Bcl-2*, *Caspase-3*, and glyceraldehyde-3-phosphate dehydrogenase *(GAPDH)* (Table 1). *GAPDH* was used as the reference gene. The specificity of each primer pair was confirmed using melting curve analysis of PCR products. The thermal cycling profile for the first round of PCR was initial denaturation and polymerase activation for 10 min at 95°C, followed by 40 cycles of 15 s at 95°C, 30 s at 58°C, and 30 s at 72°C. The final extension was for 10 min at 72°C. All reactions were performed on a Step One Plus instrument (Applied Biosystems). The 2-ΔΔCt method was used to transform Ct values into normalized values for relative expression levels.

**Table 1:** Primer pairs used for real-time PCR.

Target gene	Primer sequence (5′-3′)	Sense (S), antisense (AS)	GenBank accession No.
*GAPDH*	GAACGGATTTGGCCGTATTG	S	GU214026.1
	GTGAGTGGAGTCATACTGGAAC	AS	
*BCL-2*	GGATAACGGAGGCTGGGATG	S	NM_009741.5
	CCAGGCTGAGCAGGGTC	AS	
*BAX*	CCAGGGTGGCTGGGAAG	S	NM_007527.3
	CACAGTCCAAGGCAGTGGG	AS	
*Caspase-3*	ACATGGGAGCAAGTCAGTGG	S	XM_017312543.3
	CGTCCACATCCGTACCAGAG	AS	

### Ultrastructural analysis

Ovary fragments were fixed overnight in a solution containing 2.5% glutaraldehyde and 4% paraformaldehyde in 0.1 M cacodylate buffer. After fixation, the samples were washed twice in the same buffer and post-fixed in a solution containing 1% osmium tetroxide, 2 mM calcium chloride, and 0.8% potassium ferricyanide in 0.1 M cacodylate buffer, pH 7.2, dehydrated in acetone and embedded in Embed 812. Polymerization was performed at 60°C for 3 days. Ultrathin sections were collected on 300-mesh nickel grids, counterstained with 5% uranyl acetate and lead citrate, and examined using a FEI Morgagni 268D (Germany) transmission electron microscope (25).

### Statistical analysis

The statistical analyses were performed using the software GraphPad Prism (8.0, USA). The chi-squared test was used to evaluate the percentages of normal follicles, as well as those of primordial and developing follicles. Data of collagen fiber distribution and stromal cell density passed the normality test and were analyzed by analysis of variance (ANOVA) and Tukey test. The data of mRNA levels for *Caspase-3, Bax,* and *Bcl-2* were analyzed by the Kruskal-Wallis test, followed by Dunn's multiple comparisons test. The results are reported as means±SE. Differences were considered significant when P<0.05.

## Results

### Protective effect of *C. racemosa* extract against the deleterious effects of dexamethasone in mouse ovaries

**Figure 1:**
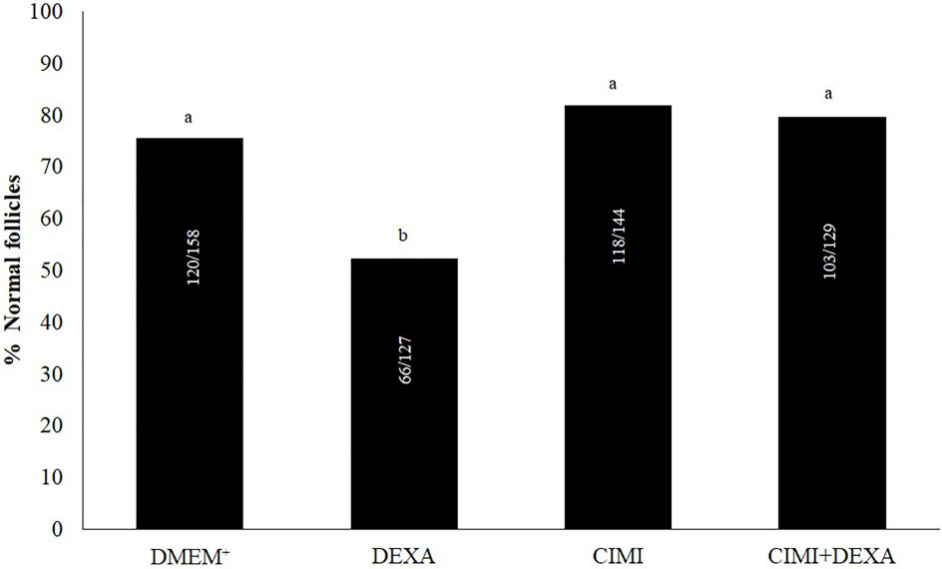
Percentage of normal follicles after 6 days of culture in DMEM^+^ alone or DMEM^+^ supplemented with dexamethasone (DEXA (4 ng/mL), *Cimicifuga racemosa (L.) Nutt* extract (CIMI) (5 ng/mL), and both CIMI and DEXA. Different lowercase letters indicate statistically significant differences between treatments (chi-squared test, P<0.05).

**Figure 2:**
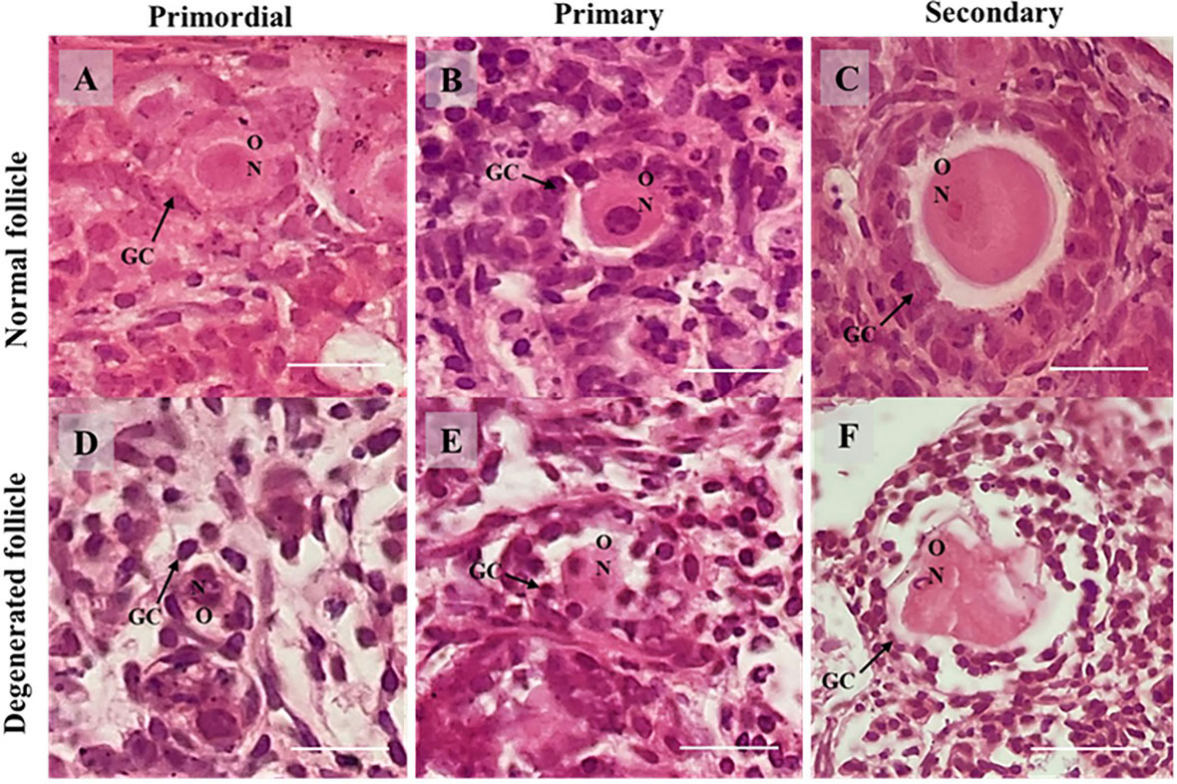
Representative images of cultured mouse ovaries showing morphological analysis. **A**, Normal and (**D**) atretic primordial follicle; **B**, Normal and (**E**) atretic primary follicle; **C**, Normal and (**F**) atretic secondary follicle. GC: Granulosa cells; O: Oocyte; N: Oocyte nucleus. Scale bar: 100 μm (400×).

The ovaries cultured in the presence of DEXA had a reduced number of normal follicles compared to those cultured in control medium alone or supplemented with CIMI or both CIMI and DEXA. No significant differences (P>0.05) were observed in the percentages of normal follicles among ovaries cultured in control medium alone or supplemented with CIMI or both CIMI and DEXA (Figure 1). The morphology of follicles from ovaries cultured in the different treatments is shown in Figure 2. Moreover, the presence of DEXA, CIMI, or both CIMI and DEXA in culture medium did not influence the population of primordial (MEM^+^: 34.2% [41/120], DEXA: 27.3% [18/66], CIMI: 36.4% [43/118], CIMI + DEXA: 30.1% [31/103]) and development follicles (MEM^+^: 65.8% [79/120], DEXA: 72.7% [48/66], CIMI: 63.6% [75/118], CIMI + DEXA: 69.9% [72/103]).

### Evaluation of extracellular matrix after *in vitro* ovarian culture

**Figure 3:**
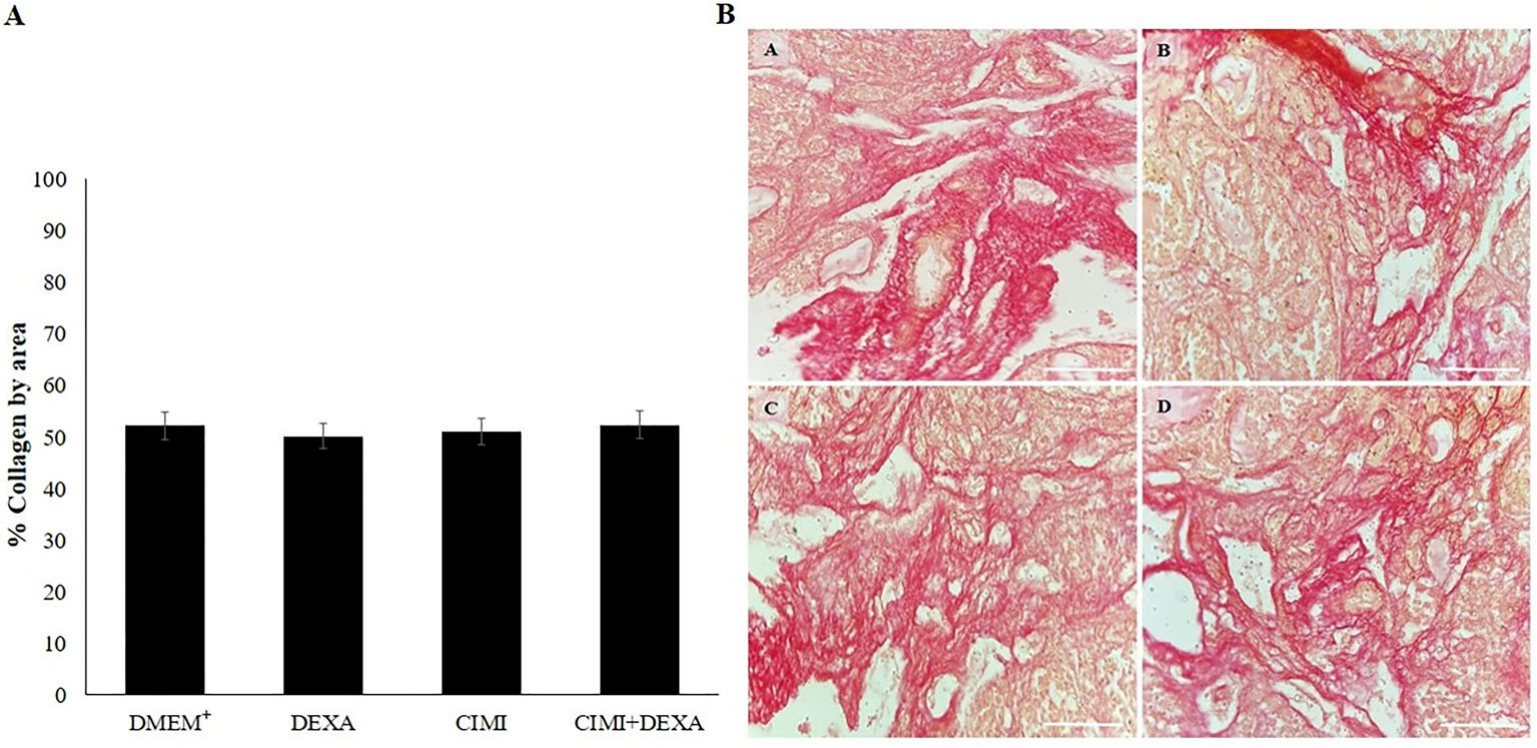
Collagen fiber levels in ovaries cultured in DMEM^+^ alone or DMEM supplemented with dexamethasone (DEXA (4 ng/mL), *Cimicifuga racemosa (L.) Nutt* extract (CIMI) (5 ng/mL), and both CIMI and DEXA (**A**). Data are reported as means±SE and were analyzed by ANOVA and Tukey test (P>0.05). **B**, Representative images of collagen fibers labeled by picrosirius red in ovaries cultured in DMEM^+^ alone (**A**), DEXA (**B**), CIMI (**C**), and CIMI+DEXA (**D**). Scale bar: 100 μm (400×).

Picrosirius red analysis showed no statistical difference in the percentage of collagen fibers between ovaries cultured in the different treatments (Figure 3A). Therefore, there was no significant damage to ECM, independent of treatment (Figure 3B).

### Evaluation of stromal cell density after *in vitro* ovarian culture

**Figure 4:**
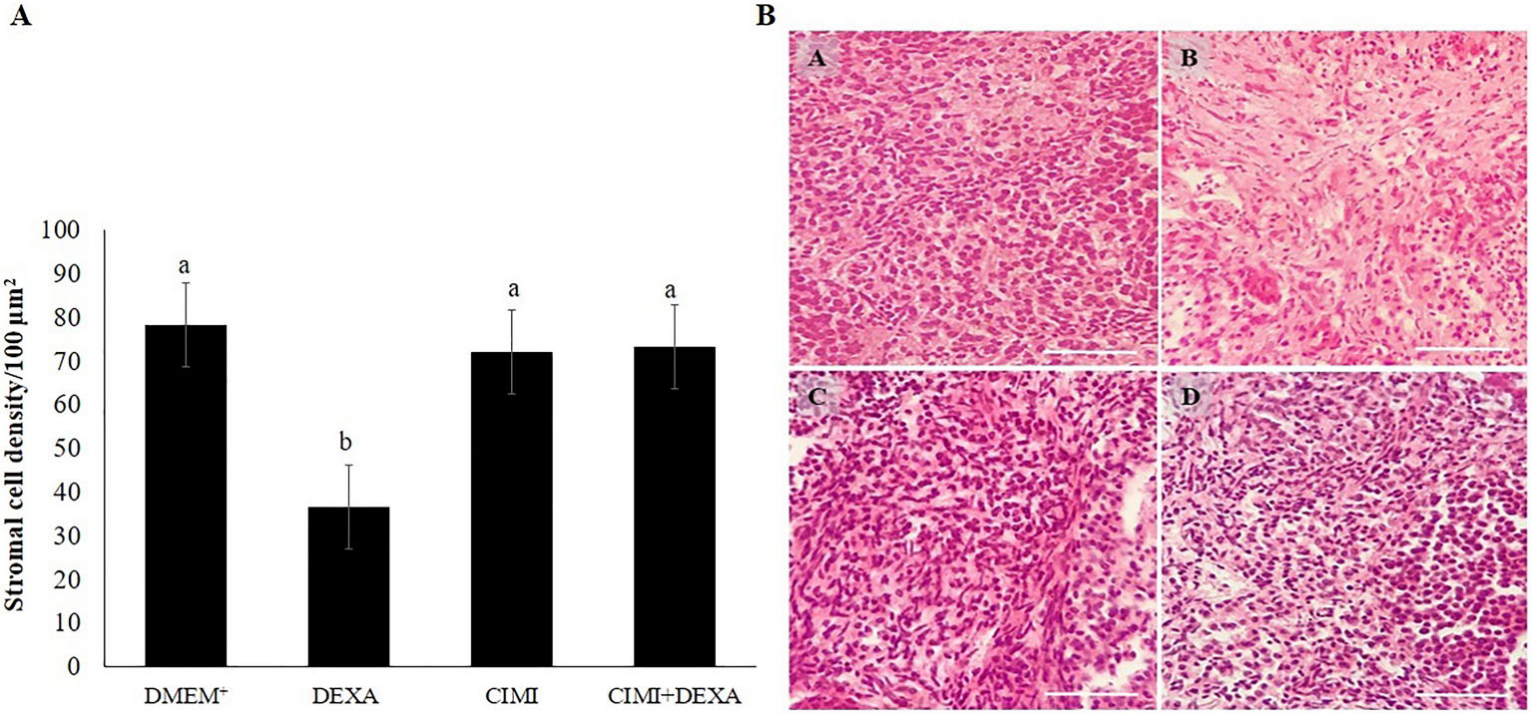
Stromal cell density. **A**, Number of cells (mean±SE) in ovarian tissue after culture in DMEM^+^ alone or with dexamethasone (DEXA), *Cimicifuga racemosa (L.) Nutt* extract (CIMI), and both CIMI and DEXA. Different lowercase letters indicate statistically significant differences between treatments (ANOVA and Tukey test, P<0.05). **B**, Representative images of stromal cell density of ovarian tissues cultured in DMEM^+^ alone (**A**) or supplemented with DEXA (4 ng/mL) (**B**), CIMI (5 ng/mL) (**C**), and CIMI+DEXA (5+4 ng/mL) (**D**). Scale bar: 100 μm (400×).

The presence of DEXA in culture medium significantly reduced the stromal cell density in the ovaries after 6 days of culture compared to ovaries cultured in DMEM^+^ alone or supplemented with CIMI. The presence of CIMI protected stromal cells against DEXA-induced damages (Figure 4A). However, when all treatments were compared with control medium (DMEM^+^), it was possible to see a reduction in the stromal cell density. Figure 4B shows histological sections of ovaries cultured in control medium alone or supplemented with DEXA, CIMI, or both.

### mRNA levels for *Bax*, *Bcl-2*, and *Caspase-3* in cultured ovaries

**Figure 5:**
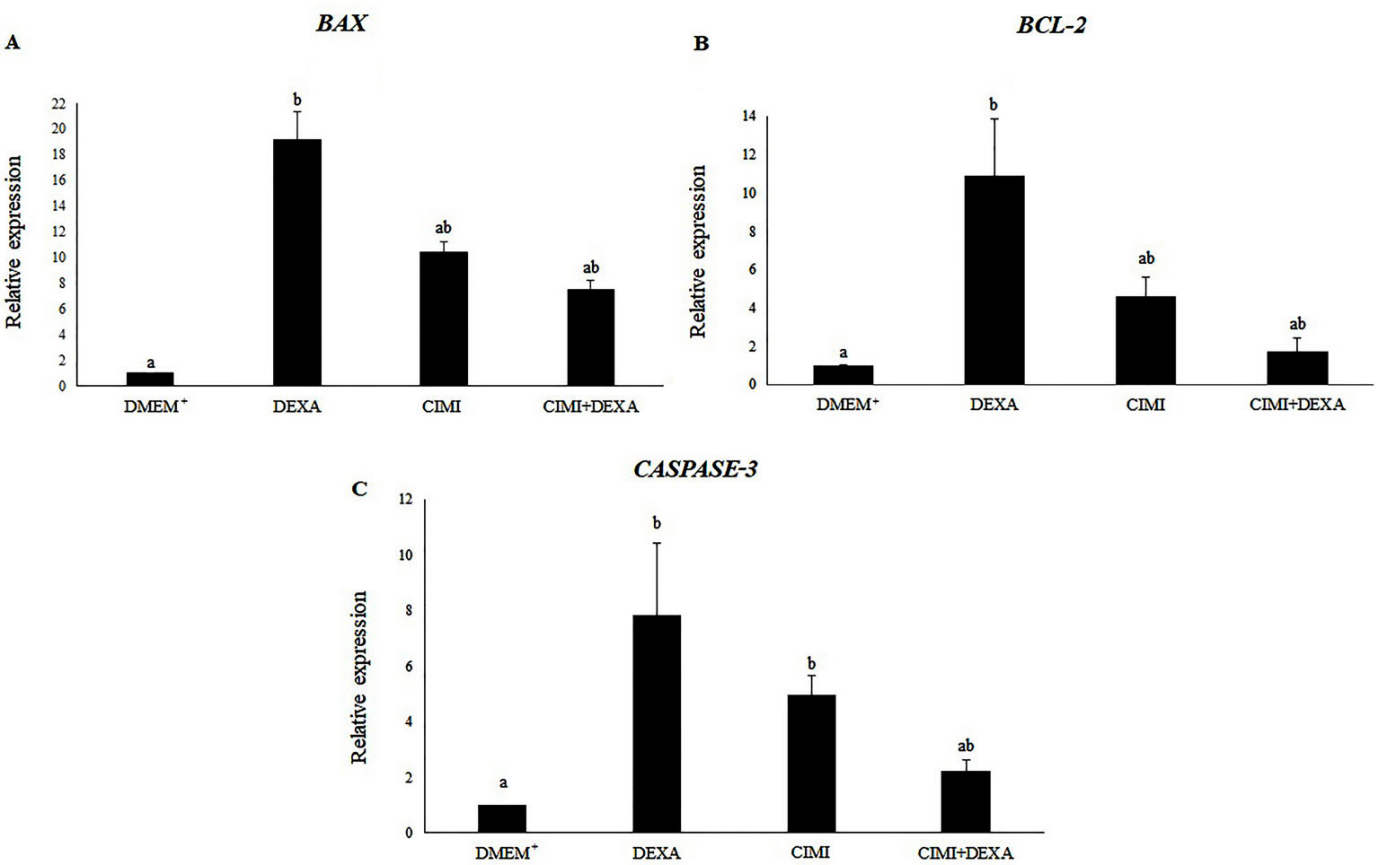
mRNA levels (means±SD) of *Bax* (**A**), *Bcl-2* (**B**), and *Caspase-3* (**C**) in mouse ovaries cultured for 6 days in DMEM^+^ alone or supplemented with dexamethasone (DEXA), *Cimicifuga racemosa (L.) Nutt* extract (CIMI), and both CIMI and DEXA. Different lowercase letters indicate statistically significant differences between treatments (Kruskal-Wallis test, followed by Dunn's multiple comparisons test, P<0.05).

Figure 5 shows mRNA levels for *Bax, Bcl-2*, and *Caspase-3* in mouse ovaries cultured *in vitro* for 6 days. The results showed that ovaries cultured with DEXA had increased mRNA levels for *Bax* and *Bcl-2*, while ovaries cultured with CIMI alone or both DEXA and CIMI had similar mRNA levels for *Bax* and *Bcl-2* compared to those observed in ovaries cultured in control medium. Additionally, there was an increase in mRNA expression for *Caspase-3* in ovaries cultured with CIMI or DEXA. On the other hand, the presence of both CIMI and DEXA kept the levels of mRNA for *Caspase-3* similar to those seen in the control group (P>0.05). However, ovaries cultured with both CIMI and DEXA had mRNA levels for *Bax, Bcl-2*, and *Caspase-3* similar to those cultured with CIMI alone.

### Ultrastructural analysis after *in vitro* culture of mouse ovaries

**Figure 6:**
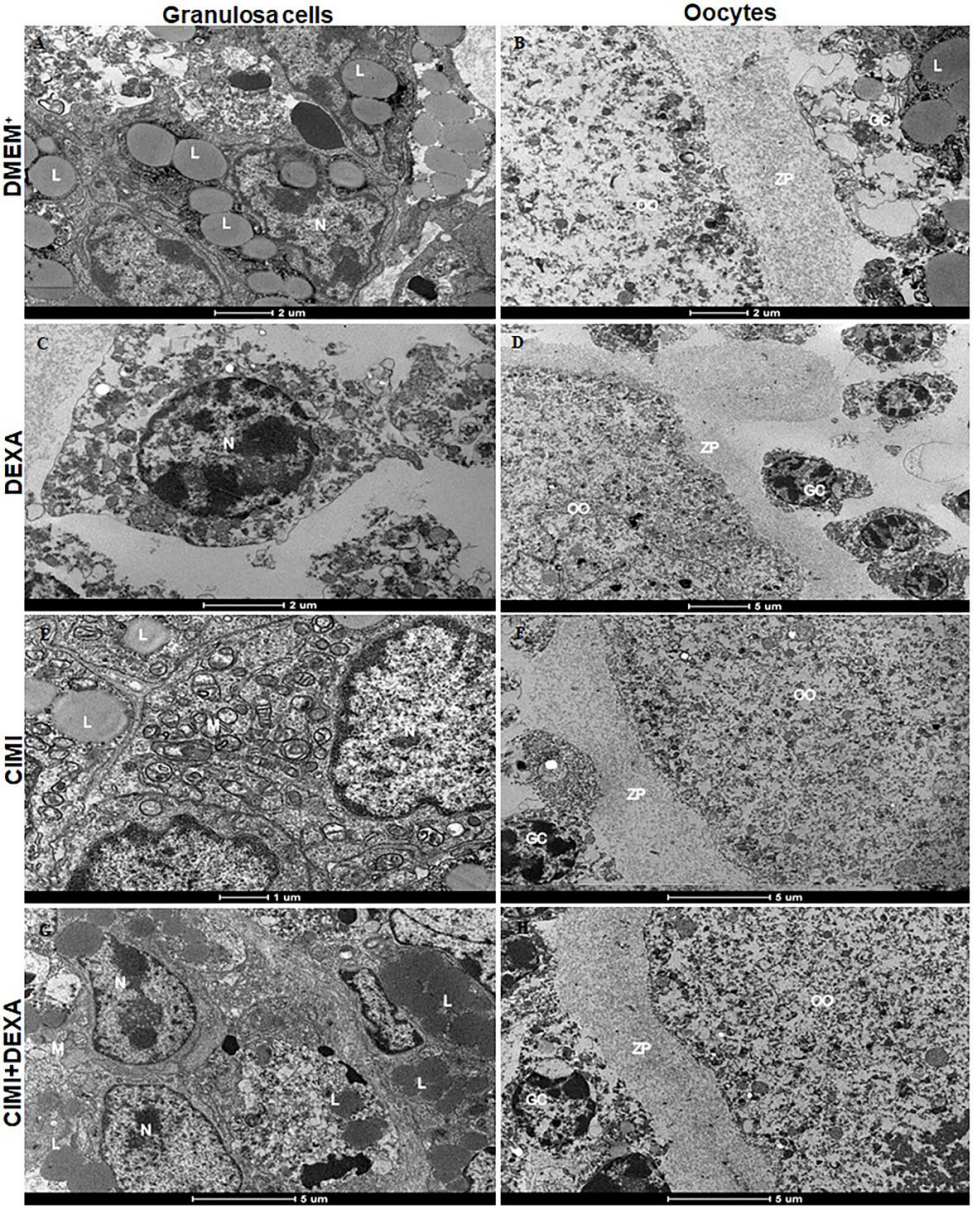
Representative micrographs of mouse ovaries cultured for 6 days in DMEM^+^ alone (**A** and **B**) or supplemented with dexamethasone (DEXA) (4 ng/mL) (**C** and **D**), *Cimicifuga racemosa (L.) Nutt* extract (CIMI) (5 ng/mL) (**E** and **F**), and CIMI (5 ng/mL) + DEXA (4 ng/mL) (**G** and **H**). M: mitochondria; N: nucleus; ZP: zona pellucida; L: lipid; OO: ooplasm; GC: granulosa cells. Scale bars: panels A, B, and C: 2 μm; panels D, F, G, and H: 5 μm; panel E: 1 μm.

Ovaries cultured in the presence of DEXA showed that preantral follicles had granulosa cells with loss of cytoplasmic integrity, organelle destruction, and increase in cellular heterochromatin regions, suggesting an initial cell death process (Figure 6C). On the other hand, ovaries cultured in control medium alone or supplemented with CIMI showed preantral follicles with well-preserved granulosa cells and mitochondria with well-organized cristae (Figure 6E). In addition, it is possible to observe the presence of lipid inclusions in granulosa cells of ovaries cultured in control medium alone or supplemented with CIMI alone or both DEXA and CIMI (Figure 6A, E, G). Oocytes from preantral follicles in ovaries cultured in control medium or supplemented with CIMI or both CIMI and DEXA showed an intact zona pellucida, well-delimited nuclear membrane, and poorly developed organelles (Figure 6B, F, H). On the contrary, ovaries cultured with DEXA had disorganized granulosa cells and oocytes with changes in their shape (Figure 6D).

## Discussion

The present study showed for the first time that CIMI extract attenuates the damages induced by DEXA in ovaries of mice cultured *in vitro*. The damage caused in the ovarian structure when cultured with DEXA may be due to overproduction of reactive oxygen species (ROS) (26). In cultured ovaries, there is evidence that ROS induces mitochondrial apoptosis pathways through the activation of cell death receptors on the membrane (27). In our study, CIMI maintained the rates of morphologically normal follicles and protected the ovarian stromal cells against DEXA-induced cytotoxicity. CIMI reduced tissue injuries induced by DEXA and maintained the ovarian stromal cell density and follicular morphology and ultrastructure. These effects probably were due to the phytochemical properties and antioxidant and anti-inflammatory activities of the extract of this plant (16,28). Previously, Rabenau et al. (17) showed that CIMI preserves mitochondrial integrity and ATP levels and prevents mitochondrial ROS formation, loss of mitochondrial membrane potential, and cell death.

Our results showed that DEXA negatively affected the stromal cell density, which is in accordance with studies carried out in human fetal ovaries (29). Additionally, DEXA is a potent inhibitor of mouse hippocampal progenitor cell proliferation (30). In the present study, CIMI alone or combined with DEXA was maintained the density of ovarian stromal cells. It is important to note that in mouse ovaries with an abnormal stromal cell organization, the proliferation of granulosa cells from secondary follicles is impaired (31). CIMI extract increased the expression of Ki-67, an indicator of cell proliferation, in the granulosa cell layer (16). CIMI was important for the maintenance of the ovarian stromal cells, which gives full support to the follicle, directly influencing follicular survival (32).

Mouse ovaries cultured with DEXA had increased mRNA levels for *Bax*, *Bcl-2*, and *Caspase-3*. The balance between apoptosis and cell proliferation during reproductive age is crucial for establishing fertility (33). Previous studies showed that DEXA stimulated the activation of caspase-3, which resulted in apoptosis of rat granulosa cells cultured *in vitro* (34). Our results showed that ovaries cultured with both DEXA and CIMI had similar mRNA levels for *Bax, Bcl-2*, and *Caspase-3* compared to those cultured in control medium alone or with CIMI. We believe that CIMI acts by reducing cellular oxidative stress and apoptosis. High rates of ROS cause irreversible cell damage, triggering a signaling program that leads to senescence, apoptosis, or necrosis (35). CIMI also had a protective effect on ovarian follicles and stromal cells, presumably through its antioxidant activity. Previously, Suh et al. (36) showed that actein, the main compound of CIMI extract, reduced oxidative stress in osteoblastic cells by increasing the activity of glyoxalase I and the levels of reduced glutathione (GSH) and transcription factor nuclear factor erythroid 2-related factor 2 (Nrf2). Recently, de Assis et al. (12) showed that CIMI also increased the mRNA levels for the enzyme superoxide dismutase (*SOD)* in mouse ovarian tissue cultured *in vitro*.

The GC-induced mitochondrial apoptotic pathway disrupts the potential of mitochondrial membranes and releases key apoptosis-inducing factors, such as cytochrome C (37). Da et al. (38) showed that CIMI inhibited the dissipation of the mitochondrial membrane potential and the oxidation of cardiolipin and decreased the release of ROS and 3-nitrotyrosine in sublingual glands of rats.

The ultrastructural analysis showed that preantral follicles of ovaries cultured with DEXA had signs of organelle destruction and increased heterochromatin regions in nuclei. Wilson et al. (39) showed that DEXA induces various ultrastructural changes in cultured cells, i.e., stacked arrangements of smooth and rough endoplasmic reticulum, proliferation of the Golgi apparatus, pleomorphic nuclei, and increased amounts of ECM material. Previously, it was reported that DEXA impaired mouse folliculogenesis and enhanced follicular atresia through induction of autophagy and apoptosis (5). In our study, CIMI helped to preserve oocytes, zona pellucida, nuclear membranes, and lipid inclusions in granulosa cells. de Assis et al. (12) recently showed that CIMI protected mouse ovarian follicles against the deleterious effects of doxorubicin.

### Conclusion

In conclusion, CIMI extract regulated mRNA expression in apoptosis, maintaining follicular ultrastructure, and protected ovarian tissue against DEXA-induced follicular atresia and changes in stromal cell density after 6 days of culture. Therefore, drugs based on CIMI can be developed to protect ovarian follicles in patients undergoing long-term treatments with glucocorticoids. Further studies are still needed to evaluate if CIMI extract interferes in the anti-inflammatory action of glucocorticoids.
